# Identification of dietary patterns using factor analysis in an epidemiological study in São Paulo

**DOI:** 10.1590/S1516-31802005000300007

**Published:** 2005-05-02

**Authors:** Dirce Maria Lobo Marchioni, Maria do Rosário Dias de Oliveira Latorre, José Eluf, Victor Wunsch-Filho, Regina Mara Fisberg

**Keywords:** Food habits, Diet, Diet surveys, Feeding behavior, Nutritional assessment, Hábitos alimentares, Dieta, Inquéritos sobre dietas, Avaliação nutricional, Conduta na alimentação

## Abstract

**CONTEXT AND OBJECTIVE:**

Diet and nutrition are environmental factors in health/disease relationships. From the epidemiological viewpoint, diet represents a complex set of highly correlated exposures. Our objective was to identify patterns of food intake in a group of individuals living in São Paulo, and to develop objective dietary measurements for epidemiological purposes.

**DESIGN AND LOCAL:**

Exploratory factor analysis of data in a case-control study in seven teaching hospitals in São Paulo.

**METHODS:**

The participants were 517 patients (260 oral cancer cases and 257 controls) admitted to the study hospitals between November 1998 and March 2001. The weekly intake frequencies for dairy products, cereals, meat, processed meat, vegetables, pulses, fruits and sweets were assessed by means of a semi-quantitative food frequency questionnaire. Dietary patterns were identified by factor analysis, based on the intake of the eight food groups, using principal component analysis as an extraction method followed byvarimax rotation.

**RESULTS:**

Factor analysis identified three patterns that accounted for 55% of the total variability within the sample. The first pattern ("prudent") was characterized by vegetable, fruit and meat intake; the second ("traditional") by cereals (mainly rice) and pulses (mainly beans); and the third ("snacks") by dairy products and processed meat.

**CONCLUSION:**

This study identified food intake patterns through an *a posteriori* approach. Such analysis may be useful for nutritional intervention programs and, after computing scores for each individual according to the patterns identified, for establishing a relationship between diet and other epidemiological measurements of interest.

## RESUMO

**CONTEXTO E OBJETIVO:**

Dieta e nutrição são fatores ambientais que interferem nas relações saúde-doença. Do ponto de vista epidemiológico, a dieta representa um complexo conjunto de exposições, altamente correlacionadas. Nosso objetivo foi realizar análise fatorial sobre dados de padrões de consumo de alimentos em um grupo de indivíduos moradores de São Paulo, e desenvolver medidas objetivas da dieta, com finalidades epidemiológicas.

**TIPO DE ESTUDO E LOCAL:**

Estudo de análise fatorial sobre dados obtidos de estudo do tipo caso-controle, em sete hospitais-escolas em São Paulo.

**MÉTODOS:**

Foram recrutados 517 pacientes (260 casos de câncer oral e 257 controles) entre novembro de 1998 e março de 2001. A freqüência semanal de consumo de laticínios, cereais, carne, carnes processadas, vegetais, leguminosas, frutas e doces foi verificada por meio de um questionário de freqüência semiquantitativo. Os padrões foram identificados por análise fatorial, a partir do consumo dos oito grupos de alimentos, utilizando a análise de componentes principais como método de extração, seguido pela rotação varimax.

**RESULTADOS:**

Foram identificados três padrões, que explicaram 55% da variabilidade total. O primeiro padrão ("prudente") foi caracterizado pelo consumo de vegetais, frutas e carne; o segundo ("tradicional") por cereais (principalmente arroz) e leguminosas (principalmente feijão); e o terceiro ("lanches") por doces, produtos lácteos e carnes processadas.

**CONCLUSÕES:**

O estudo identificou padrões de consumo de alimento, utilizando uma abordagem "*a posteriori".* Esta análise pode ser utilizada em programas de intervenção nutricional, bem como, a partir do estabelecimento de escores de indivíduos segundo os padrões identificados, para relacionar a dieta com outras medidas epidemiológicas de interesse.

## INTRODUCTION

Diet and nutrition are important factors in the promotion and maintenance of good health throughout the entire course of life. For a long period of time, the major concerns of researchers and health professionals were in relation only to the prevention of deficiency-related diseases such as scurvy and beriberi. Over recent decades, however, there has been additional concern relating to the prevention of chronic non-communicable diseases such as cardiovascular diseases, cancer, diabetes, hypertension and osteoporosis.^1^

The complexity of the human diet presents a challenge to those intending to study the relationship between diet and disease. Diet has traditionally been studied in terms of nutrients. However, it should be emphasized that foods contain other chemical compounds, some well-known, some still poorly characterized and others completely unknown and which at present cannot be measured.^2^ Furthermore, the diversity of food combinations may lead to competition, antagonism or alteration in nutrient bioavailability. From an epidemiological viewpoint, the diet represents a complex set of highly correlated exposures. Thus, the real relationship between a food group and a disease may erroneously be attributed to a single component, because of the multicollinearity that exists between nutrients and foods.^3,4^

One option when dealing with the complexity of intercorrelations between foods is the use of pattern analysis. This approach uses the correlations between food and nutrient intake to describe a general dietary pattern that at a later stage may be related to the risk of a disease. This approach is of particular value if the effect of the diet is not mediated by one or two specific nutrients, but by nutrients that perhaps operate interactively.^3,5,6^

Two approaches have been used for developing general descriptors of dietary patterns. The first approach, called *a priori,* is based on previous knowledge of the favorable and unfavorable effects of diet constituents (e.g. by using the Diet Quality Index).^7^ Another approach, *a posteriori,* is based on the dietary data obtained. The main techniques in this latter approach are principal component analysis, followed by factor analysis, and this approach requires statistical modeling.^6^ The goal is to transform a large set of correlated variables into a smaller set of non-correlated variables called principal components or factors. In factor analysis, rather than establishing a diet indicator, the data objectively point towards how measurements are clustered. The aim of this technique is to identify the underlying structure in a data matrix, by summarizing and reducing data in order to supply a synthetic measurement of the diet. In order to summarize the data, factor analysis derives dimensions that, when interpreted and understood, describe the data in terms of a much smaller number of items than do the individual variables.^8,9^

The aim of the present study was to describe the food patterns in a group of individuals who were participating in a Latin American case-control study, and to relate environmental factors to cancer of the oral cavity and larynx through the *aposteriori* approach.

## METHODS

The present study utilized data obtained from a multicenter, hospital-based case-control study^10^ that was performed with the support of the International Agency for Research on Cancer (IARC). Between November 1998 and March 2001, 517 patients were recruited, including 260 cases of cancer of the oral cavity, oropharynx and hypopharynx that had been identified in seven hospitals in the municipality of São Paulo, and 257 controls that had been admitted to these same hospitals because of conditions that were unrelated to diseases associated with risk factors for cancer of the oral cavity. The study was approved by the National Committee for Ethics in Research (Comissão Nacional de Ética em Pesquisa - CONEP).

### Dietary data

Dietary intake information was collected using a semiquantitative food-frequency questionnaire (FFQ). The FFQ list consisted of 27 foods, food groups or preparations. Each participant in the study was asked to provide, for each item of the FFQ, the mean weekly intake frequency before the emergence of disease symptoms. The answers were open, thus allowing this variable to be treated as continuous. The foods on the questionnaire were then classified into the following food groups: dairy products (milk, yogurt and cheese); cereals (bread, rice, pasta and maize); meat (beef, pork, poultry and fish); processed meat (sausage, salami and ham); vegetables (raw vegetables, cruciferous plants, tomatoes and carrots); pulses (beans and peas); fruits (apples, pears, bananas and fruit juices) and sweets (deserts, sweets and cakes). These groupings were based on the similarity of nutrient content.

### Statistical analysis

Dietary patterns were obtained by exploratory factor analysis of the eight food groups. Factor analysis is a generic name given to multivariate statistical analysis applied to the identification of factors in a set of measurements. Such factors would correspond to indicators. In this method, all variables are considered simultaneously, each one related to the others.

Initially, in order to verify the appropriateness of using factor analysis, the sample uniformity was tested by examining the distribution of the variables in a loading plot, contrasting the values observed to those expected in a normal distribution. The data adjustment was verified using the Kaiser-Meyer-Olkin (KMO) measurement of sample adequacy and the Bartlett Test of Sphericity (BTS), which tests the presence of correlations between variables.

Principal component analysis was used for factor extraction. This method studies the spatial distribution of the objects so as to identify groupings and the relationships between them. The first factor extracted is the one that accounts for the maximum possible variance in the data set. The second component, independent of the first, will be the one that explains the greatest possible share of the remaining variance, and so on, without the components being correlated with each other.^9,11^

The choice of the number of factors was first based on the Kaiser criterion, namely eigenvalues over 1.0. This is the most frequently used criterion in factor analysis, and the theoretical basis behind it is that each retained factor should explain more variance than the original variable in the data set. Factor determination was also based on the eigenvalue plot (scree plot), which plots the total variance associated with each factor.

Factor loadings, i.e. measurements of correlations between factors derived from the original measurements, were analyzed after orthogonal rotation using the varimax method.^12,13^ That is, each factor was independent of the others, maintaining the axes at 90°. This operation provided a simpler structure, through distributing the explained variance among the individual components, thus increasing the numbers of higher and lower factors. Factor loadings of more than 0.3 were considered to be contributing significantly to the factor. Within a factor, negative loadings indicated that the food group was inversely associated with the factor, while positive loadings indicated a direct association. The higher the factor loading of a food group was, the greater the contribution of that group to the factor, since the square of the factor loading corresponded to the percentage of the variance of the food group explained by the factor. The loading plot aided factor interpretation by examining the location of the variables in a system of coordinates created by the factors.

Statistical analyses were performed using Statistical Package for Social Science (SPSS) 10.0 software.

## RESULTS

Table 1 presents the distribution of cases and controls according to sociodemographic variables and smoking and drinking habits.

**Table 1:** Description of the studied population (260 cases of cancer and 257 controls) used for identifying dietary patterns

Variable	Total	Cases		Controls		χ^2^ p value
		n	%	n	%	
**Sex**						
Male	413	224	54.2	189	45.8	-*
Female	104	36	34.6	68	65.4	
**Age (years)**						
< 45	93	38	40.9	55	59.1	-*
45-54	130	78	60.0	52	40.0	
55-64	142	70	49.3	72	50.7	
> 65	152	74	48.7	78	51.3	
**Smoking status**						
Never smoked	102	14	13.7	88	86.3	< 0.01
Former smoker	143	58	40.6	85	59.4	
Current smoker	272	188	69.1	84	30.9	
**Drinking habit**						
Never drank	103	20	19.4	83	80.6	< 0.01
Former drinker	208	108	51.9	100	38.9	
Current drinker	206	132	54.1	74	35.9	
**Education** **						
Elementary school	362	185	51.1	177	48.9	0.31
High school	55	26	47.3	29	52.7	
University	18	7	38.9	11	61.1	

* *variables selected for matching*

** *information missing for 42 cases and 40 controls.*

### Dietary Components

**Figure 1:**
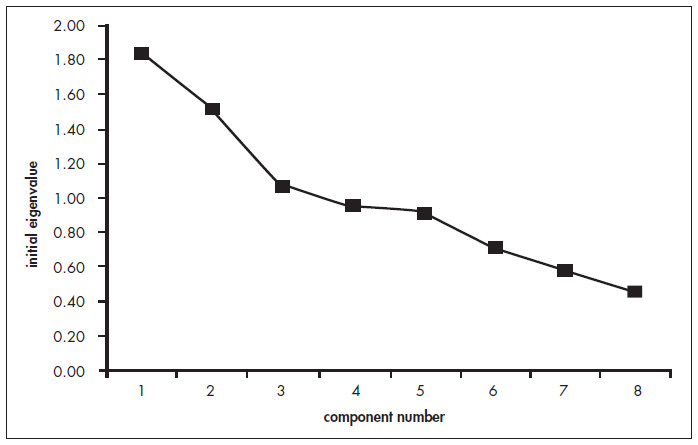
Scree plot showing *eigenvalues* for each component, in factor extraction of data obtained from a food-frequency questionnaire answered by 517 people.

The observed KMO was 0.56, which meant that the sample was considered to be adequate for factor analysis. Three components were identified through factor analysis, based on the Kaiser criterion and the scree plot (Figure 1). These three components accounted for 55% of the variability within the sample.

Table 2 shows the factor loadings obtained after varimax rotation.

**Table 2:** Food group factor loadings for the three dietary patterns identified in the data obtained by a food-frequency questionnaire answered by517 people

Food group	Dietary pattern		
	Prudent	Traditional	Snack
Dairy products	0.290	-0.195	**0.566**
Cereals	0.254	**0.812**	0.092
Meat	**0.617**	0.276	0.021
Processed meat	0.091	0.025	**0.540**
Vegetables	**0.807**	-0.133	-0.019
Pulses	-0.154	**0.850**	-0.049
Fruits	**0.651**	0.001	0.225
Sweets	-0.112	0.134	**0.744**
% Explained variance	22.9	18.9	13.4
Cumulative %	22.9	41.8	55.2

*Extraction method: principal component analysis.*

The first factor, which accounted for 23% of the total variance, was labeled *prudent*. Vegetable, fruit and meat intake characterized this factor.

The second factor explained 19% of the total variance. Since this factor was characterized by the intake of cereals and pulses, it was labeled *traditional.*. Both vegetable and dairy product groups were negatively associated with this factor.

The third factor accounted for approximately 13% of the total variance. High factor loadings were observed for sweets, dairy products and processed meat. This factor was labeled *snacks*.

**Figure 2:**
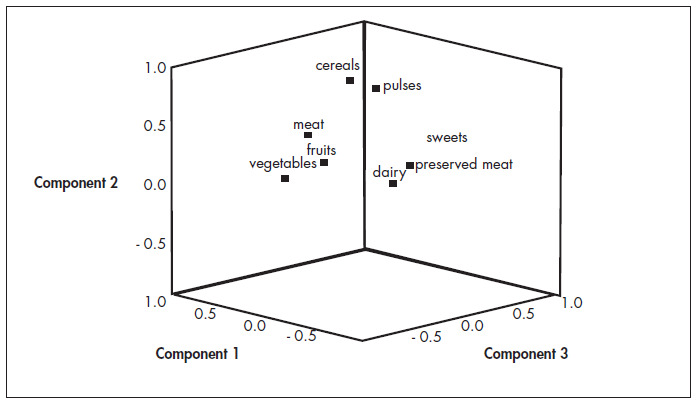
Spatial representation of the relationships between derived factors and the dietary variables of the study of the dietary patterns of 517 people.

Figure 2 shows the spatial graphical representation of the derived factors. On this graph, the groupings of variables and their relationships with the derived factors can be seen.

## DISCUSSION

Studies that identify dietary patterns in developing countries are scarce. The present analysis explores dietary patterns in a group of subjects who were participating in a case-control study in the metropolitan area of São Paulo, Brazil, using factor analysis. Three patterns, *prudent, traditional* and *snacks*, that explained 55% of the total variability, were identified.

The first factor was characterized by the presence of fruits and vegetables. The contribution of fruits and vegetables to health seems to be due to the variety of phytonutrients and the potassium and fiber contained in these foods. The second factor was characterized by the presence of pulses, which in Brazil comprise essentially beans, and cereals, especially rice. These two foods, considered to be typical of the Brazilian diet, represented the greatest communalities, i.e. the greatest contributions to the model. A previous study conducted in Rio de Janeiro also observed a pattern characterized by the predominance of rice and beans.^14^ The third pattern, in its turn, was negatively associated with vegetables and pulses and was characterized by the presence of foods that are associated with increased risk of chronic diseases. These include processed meats, which have high sodium and saturated fat content, and sweets. Diets containing carbohydrates with a high glycemic index have been associated with high fasting glucose and insulin levels, increased risk of impaired glucose tolerance (IGT) and increased IGT rates that developed to diabetes.^15^

The large numbers of highly correlated variables hinder the conducting of dietary studies, and traditional classification methods may lead to erroneous estimates. This multivariate method may represent an alternative approach to the evaluation of individual nutrients,^16^ since the identification of patterns allows us to examine the effect of the diet as a whole and to describe associations with diseases beyond those described for single nutrients or foods. The patterns identified may be used as co-variables in order to determine whether the effect of a specific nutrient is independent of the dietary patterns.^17-19^

Moreover, it should be borne in mind that individuals consume nutrients based on their food choices, which are influenced by a variety of cultural, social and demographic factors. Describing food intake in patterns may be particularly useful in developing counseling programs. Rather than changing the nutrient intake, such programs can be aimed at changing the intake of foods that are readily recognized by the target group.^20^ At present, dietary guidelines are published with emphasis on foods and overall dietary patterns.^15^

The patterns extracted in this study differed from those found in earlier studies on adult populations.^5,16-21^ It should be emphasized that patterns are considered to be comparable only if the food groups and the factor loadings relating to their magnitudes are similar. In fact, since the patterns are extracted from the data obtained in the studied population, it is not surprising that the results are not reproduced in populations with different food habits. However, this characteristic of the method may lead to difficulties in reproducing risk estimates in different study populations.

In this approach, the researcher has to make decisions and establish judgments at several stages of the process, which may yield biased results regarding the selection of the variables that participate in the analysis, the number of retained factors and factor interpretation.^17,22^ In the present study, the variables were grouped by similarity in terms of composition and nutrient value, based on previous studies using similar criteria.^17,18,20^ Furthermore, the three retained factors explain more than half of the total variance. According to Schulze et al. (2001),^19^ if the patterns fail to explain much of the variance in the food intake as a whole, it is possible that these patterns would not explain much of the variance in a single food or nutrient either, thus limiting their use in nutritional epidemiology. However, it is inappropriate to analyze the relationship between nutrients and disease through dietary pattern analysis, since this analysis is not specific for such purposes. Pattern analysis may be useful when the traditional approach, which is focused on nutrients, identifies only a small number of specific associations with the disease.^18^

## CONCLUSION

Our data allowed the identification of dietary patterns defined via factor analysis, based on data from a food-frequency questionnaire. It is now important to demonstrate the link between specific patterns and health status. Once these links are clearly defined, it will be possible to develop nutritional interventions based on these patterns.
